# Formulation, Characterization and Evaluation of Innovative O/W Emulsions Containing Curcumin Derivatives with Enhanced Antioxidant Properties

**DOI:** 10.3390/antiox11112271

**Published:** 2022-11-17

**Authors:** Evdokia Dalla, Ioanna Koumentakou, Nikolaos Bikiaris, Evangelia Balla, Smaro Lykidou, Nikolaos Nikolaidis

**Affiliations:** Laboratory of Chemistry and Technology of Polymers and Dyes, Department of Chemistry, Aristotle University of Thessaloniki, 54124 Thessaloniki, Greece

**Keywords:** oil in water, emulsions, curcumin, β-cyclodextrin, curcumin extract, antioxidants, antimicrobial

## Abstract

In the present study, a series of semisolid Oil in Water (O/W) emulsions containing different Curcumin (Cur) derivatives (Cur powder, Cur extract and Cur complexed with β-cyclodextrin) in varying concentrations, were prepared. Initially, Dynamic Light Scattering (DLS), microscopy, pH and viscosity measurements were performed to evaluate their stability over time. Moreover, the effect of the active cosmetic substances on the Sun Protection Factor (SPF), antimicrobial and antioxidant properties of the prepared emulsions was investigated. It was observed that emulsions containing Cur extract and Cur β-cyclodextrin complex presented great viscosity and pH stability for up to 90 days of storage contrary to the emulsions containing Cur powder which showed unstable behavior due to the formation of agglomerates. All samples presented SPF values between 2.6 and 3.2. The emulsions with Cur in all forms exhibited high antioxidant activity, whereas the emulsion containing Cur β-cyclodextrin complex presented the highest value. Despite their improved stability and antioxidant activity, the emulsions containing Cur extract and Cur-β-cyclodextrin exhibited a low percentage of antimicrobial activity against *E. coli* and *Staphylococcus* bacteria. Instead, the emulsions containing Cur powder presented a reduction rate over 90 % against *E. coli* and *Staphylococcus* colonies.

## 1. Introduction

Human skin’s exposure to solar ultraviolet radiation (UVR) leads to a dramatic increase in the production of reactive oxygen species (ROS), thus shifting the natural balance towards a pro-oxidative state and resulting in oxidative stress [1]. In turn, the oxidative stress induces undesirable and deleterious skin diseases, such as aging, scaling, dryness, mottled pigment, and, ultimately, skin cancer, the most severe consequence of photodamage [2]. These multiple detrimental effects are caused by a series of photochemical reactions, such as changes to the DNA sequence, oxidation of nucleic acids and modification of proteins and lipids resulting in functional alterations. Therefore, regulation of ROS levels is critical to the maintenance of normal skin homeostasis [3].

The body is well equipped with antioxidant enzymes that deal with the oxidative stress and resist to these changes [2]. However, UVR and other free-radical generators (e.g., cigarette smoke or outdoor air pollution) can overwhelm the system, rendering the natural antioxidant compounds inadequate to protect the skin from the oxidative damage. Thus, cosmetic science has been receiving increasing attention towards the development of products with effective antioxidant agents [1]. Topically applied antioxidants constitute an important group of pharmacologically active agents capable of inhibiting the oxidation of other molecules, thus protecting the skin cells against the damaging effects of ROS [3]. Lately, studies upon artificial antioxidants, unfortunately, exhibited dose-dependent toxicological effects. Therefore, the search for new natural-derived antioxidants is crucial [4,5]. Several natural topical antioxidants have been already investigated, such as vitamin C [3], tea catechins [6], cinnamon leaf oil, table grapes [7], hippophaerhamnoides [8] and others. However, the majority of them are very unstable, since they are easily oxidized and consequently lose their activity before reaching the target.

Lately, curcumin has arisen as an ideal candidate among other natural-derived components, due to its numerous phenolic groups leading to beneficial effects in several human disorders and its low toxicity [9]. Curcumin (Cur) is a natural occurring lipophilic polyphenol located in the rhizomes of turmeric, also known as Curcuma longa, which has received recognition as a bioactive compound with great antioxidant properties. Although orally ingested Cur may exhibit potential health benefits when received at sufficiently high levels, it is quite challenging to directly incorporate it into many pharmaceutical formulations and supplement products due to its low oral bioavailability, high chemical instability, and poor water-solubility [10]. To improve its bioavailability of curcumin, various approaches have been reported by researchers including the synthesis of liposomal curcumin [10], nanocurcumin [11], curcumin–phospholipid complex [12], and chelation with metals [13]. Solubility issues can be also overcome by the formation of inclusion complexes with cyclodextrins, namely, cyclic oligosaccharides formed by non-reducing chiral glucose-building blocks connected with a ring structure [14]. These systems not only improve Cur solubility and stability but also deliver Cur in its active form. Among such carriers, β-cyclodextrin (β-CD) is a widely used cyclic bucket-shaped oligosaccharide. It is a semi-natural product with extremely low toxicity that enhances drug delivery through biological membranes [15].

An alternative approach to overcome such challenges is the design and fabrication of emulsion-based delivery systems, the composition and structure of which must be optimized for their appropriate utilization [16,17]. Emulsions are thermodynamically unstable systems that tend to gradually destabilize or can be immediately destructed during storage, under the effect of temperature and/or by the addition of another compound (e.g., electrolytes, bioactives, etc.) [18]. They are prepared during emulsification, a special mixing process based on the supply of energy under stirring of two immiscible liquids phases (oil and water), in order to disperse each other in the form of droplets, and they are stabilized by emulsifiers, thickening and jelly agents, etc. This results in a stable system. In oil-in-water emulsions, the continuous or external phase is the water, while the discontinuous or internal phase is the oil. A variety of proteins or polysaccharides, such as soy carbohydrates or casein, whey protein, Arabic gum, lecithin or Tween-80, are typically used as surfactants [14]. 

In the present work, Polysorbate 60 was used as a surfactant and emulsifying agent. It is a hydrophobic nonionic surfactant that is derived from vegetable oil. In cosmetics, its main function is to enhance the emulsification process and lubricity of emulsions [19].

Cetearyl and cetyl alcohols were also used as surfactants and emulsifiers to stable the emulsions, holding water and oil together [20]. 

For the formulation of the oil phase, the natural ingredients shea butter, olive oil and beeswax were used to give a soothing feeling, to emollient the skin and to maintain the skin hydration [21,22,23]. Glycerol was added to the water phase to enhance the solubility of the non-polar oil molecules in the surrounding aqueous phase since a solution containing glycerol is less polar than pure water [24]. Citric acid was used as an emulsifying agent, for the stabilization of the emulsions, as well as performing as a pH regulator [25]. Additionally, Xanthan gum was added to improve the stability of the prepared system and control the creaming phenomenon which is essential in a cosmetic emulsion [26]. 

The current study has focused on the synthesis of O/W emulsions containing three different Cur derivatives (powder, extract, and β-CD complex) to produce stable cosmetic emulsions with excellent antioxidant properties. Cur extract and Cur-β-cyclodextrin complex were implemented to improve the bioavailability, solubility, and stability of Cur powder. The antioxidative, antimicrobial, SPF and stability behavior of all prepared emulsions were investigated in vitro, and the results of emulsions were compared to each other. The innovation in this work lies in the formulation of emulsions with enhanced antioxidant and antimicrobial properties using natural-Cur derivatives components, avoiding drugs that may cause some side effects in the human organism and maintaining the emulsion stability.

## 2. Materials and Methods

### 2.1. Materials

Cur powder (p-Cur) was purchased from Avramoglou Spices and Aromatic Plants (Edessa, Greece), and the curcumin-β-cyclodextrin complex (b-Cur) was purchased from Wacker Chemical (München, Germany).The curcumin extract (e-Cur) was received after steam distillation of p-Cur at 60 °C using a water bath. For the preparation of O/W emulsions, olive oil, sesame oil, ethylhexylglycerin, shea butter, glycerin, cetostearyl alcohol, cetyl alcohol, sodium citrate, beeswax, xanthan gum, polysorbate 60, steatic acid, triglycerides and phenoxyethanol were kindly donated from Novita Group (Thessaloniki, Greece). All other materials and reagents used in this study were of analytical grade of purity.

### 2.2. Preparation Methods of the Emulsions

**Figure 1:**
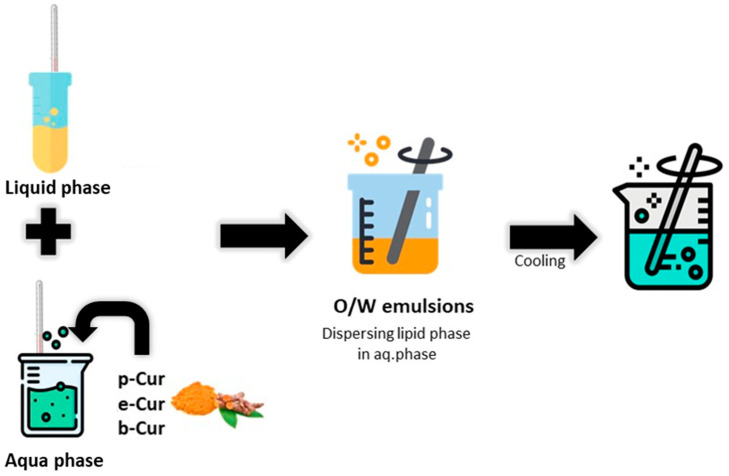
Experimental process for the preparation of p-Cur, e-Cur and b-Cur O/W emulsions.

The preparation of the emulsions was conducted according to O/W (oil-in-water) technique followed in previous work [4,27] (Figure 1). Table 1 summarizes the utilized substances for the emulsions’ fabrication. The water phase (75%) consisted of water (140 g, 70% *w*/*v*), glycerin (7 g, 3.5% *w*/*v*), xanthan gum (2 g, 1% *w*/*v*) and citric acid (1 g, 0.5% *w*/*v*). The water phase was heated in a water bath at 80 °C until xanthan gum was completely dissolved, and it was then homogenized, using a 2020 RZR (Heidolph, Schwabach, Germany) mechanical stirrer at 600 RPM. The bioactive substances were added in various weight ratios in the water phase. More specifically, p-Cur was added in and 0.5 and 2 % *w*/*v* ratios, e-Cur in 1% *w*/*v* ratio and b-Cur in 1% *w*/*v* ratio. Equal ratios of water were removed in each emulsion from the water phase. The oil phase (25%) was consisted of olive oil (26 g, 13% *w*/*v*), cetyl alcohol (4 g, 2% *w*/*v*), cetostearyl alcohol (4 g, 2% *w*/*v*), polysorbate 60 (4 g, 2% *w*/*v*), shea butter (4 g, 2% *w*/*v*), steatic acid (4 g, 2% *w*/*v*) and beeswax (4 g, 2% *w*/*v*). The oil phase was heated until a homogenous solution was received, was then added dropwise to the water phase in O/W ratio 25/75 ratio under continuous mechanical stirring. The produced O/W emulsions were left under mechanical stirring (400 RPM) for 2 h and then phenoxyethanol and ethylhexylglycerin preservatives were also added. Finally, a blank emulsion without the addition of any bioactives was also prepared according to the procedure described above. Final volume/weight ratio of the produced emulsions was 75% (150 mL/ 200 g).

### 2.3. Optical Microscope

Optical microscope Leica (Leica Microsystems Wetzal GmbH, Germany) equipped with image analysis system (Leica Q5001 System, Leica Cambridge, UK) consisting of color image capture device (JVC Colour Digital Camera, TKC1381, Yokohama, Japan) was used for the microscopic examination of the prepared emulsions. During the examination samples are taken from each prepared emulsion and their observation is made at intervals of 0, 30, 60 and 90 days.

### 2.4. Dynamic Light Scattering (DLS)

DLS technique was used for the measurement of the droplets size of the prepared emulsions. A 100 µL sample of each emulsion was dispersed in 900 µL of double distilled water in 2000 rpm, 56.4 laser power and measured size may range of 0.3 nm to 10 um. (Zetasizer 5000, Malvern Instruments, Worcestershire, UK). All measurements were performed in triplicate [4].

### 2.5. pH and Viscosity Stability

The stability of the prepared emulsions was studied via pH and viscosity measurements after 1, 7, 14, 30, 60 and 90 days after the preparation of the emulsions. The pH value was assessed by dipping the pH sensor (Microprocessor, WTW, pH 535, Gemini BV, Apeldoorn, The Netherlands) in the emulsions. Viscosity measurements were performed using the R3 spindle of a Visco Star Plus viscometer under 50 and 100 rpm [27]. 

### 2.6. Sun Protection Factor (SPF) 

For the SPF measurements, diluted solution transmittance method was used. Next, 1% *w*/*v* each sample was added in ethanol, sonicated until complete homogenization and filtered through Whatman filters. 20% *v*/*v* of each solution was diluted with ethanol. The absorption values of the emulsions were achieved in the range of 290–320 nm (every 5 nm) using a UV-Vis spectrophotometer (Shimadzu, Tokyo, Japan) [28]. The experiments for each formulation were achieved in triplicate. Mansur equation was utilized to determine the SPF values of the emulsions:

(1) $$\mathrm{SPF}\;\mathrm{in}\;\mathrm{vitro}\;=\mathrm{CF}\;\times \sum_{290}^{320}\mathrm{EE}\left(\lambda \right)\times I\left(\lambda \right)\times \mathrm{abs}(\lambda )$$

where CF is 10 (correction factor), EE(λ) is the erythemogenic effect of radiation at wavelength λ, I(λ) denotes the intensity of solar light at wavelength λ and abs(λ) denotes the absorbance of the sample at wavelength λ. The values for the term “EE × I” are constants, which were determined by Sayre et al. [29].

### 2.7. Antioxidant Study

The antioxidant activity of the samples was determined via the 2,2-Diphenyil-1-picrylhydrazyl (DPPH) method, according to Blois method developed in 1958 [30]. A volume of 1 mL of each emulsion dispersed in EtOH (1% *v*/*v*) was added to 3 mL of a 5 × 10^−3^ mg/mL ethanol DPPH solution. The reference sample composed of ethanol and the DPPH/EtOH solution in 1/3 ratio. The samples were sonicated for 10 min, and their absorbance was recorded 30 min later at 517 nm with the aid of a UV-Vis spectrometer (UV Probe 1650, Shimadzu, Tokyo, Japan). The free radical scavenging activity was described as described by Brand et al. according to the following equation [31]:

(2) $$\mathrm{Free}\;\mathrm{radical}\;\mathrm{scavenging}\;\mathrm{activity}\;\left(\%\right)=\frac{\mathrm{Absorbance}\;\mathrm{of}\;\mathrm{control}-\mathrm{Absorbance}\;\mathrm{of}\;\mathrm{extracts}}{\mathrm{Absorbance}\;\mathrm{of}\;\mathrm{control}}\times \;100$$

### 2.8. In Vitro Antimicrobial Properties

Antimicrobial activity was determined against two different bacteria, negative Gram *Escherichia coli* (BL21) and positive Gram *Staphylococcus aureus* (ATCC 25923). Bacteria were grown in 100 mL of sterile medium Luria–Bertani (LB) under 150 rpm stirring. The cells were then harvested at the logarithmic growth phase and the concentration of their suspensions was adjusted to OD600 (optical density at 600 nm) at 0.5% *w*/*v* with an appropriate amount of 25 mM PBS buffer (Sigma Aldrich, Saint Louis, MO, USA), before incubating the cells with each emulsion. Approximately 500 mg of each emulsion were placed in sterilized plates of multiple seats (12 seats) and were incubated with 1 mL of the cell suspension at 37 °C under mild stirring for 16 h. Removal of the bacterial suspension, three washes of the emulsions with LB, and removal of the cells that were in contact with the emulsions using 1 mL of TAT (Trypton-Azolectin-Tween) solution were then followed. The density of each bacterial culture on each emulsion was quantified by measuring the colonies that developed, by the method of sequential dilutions. More specifically, to determine the antibacterial activity, 100 μL of the cell suspensions obtained with TAT wasdiluted sequentially with LB medium to obtain 10^−1^, 10^−2^, 10^−3^, 10^−4^ and 10^−5^ dilutions. A volume of 100 μL from each plate was combined with 900 mL of LB agar as medium. Dilutions assist to the measurement of the colonies at each sample. The resulting colonies after 24 h of incubation at 37 °C were counted and the number of colony forming units per μL was calculated (CFU/μL). The blank cream, to which no active substance was added, was used as a control standard. Number of colonies cultivated in each plate was measured using Analytikjena device.

The number (No) of bacteria recovered is calculated from the following equation:

(3) No of bacteria recovered =No of bacteria on plate × TAT (mL) × Dilution factor

The percentage of reduction in bacteria for each sample of emulsion (% R) is calculated from following equation:

(4) $$\%\;\mathrm{Reduction}\;=\frac{\left(\mathrm{No}\;\mathrm{of}\;\mathrm{bacteria}\right)\mathrm{blank}\;\mathrm{emulsion}-\left(\mathrm{No}\;\mathrm{of}\;\mathrm{bacteria}\right)\mathrm{sample}}{\left(\mathrm{No}\;\mathrm{of}\;\mathrm{bacteria}\right)\mathrm{blank}\;\mathrm{emulsion}}$$

Higher values of %R of bacteria reduction in each sample leads to higher antimicrobial activity. 

LC-HRMS Orbitrap Analysis was developed and validated for the determination of curcumin content in all the prepared emulsions aiming to enhance the results of the antioxidant and antimicrobial properties and associate them with the presence of phenolic compounds. 

## 3. Results and Discussion

### 3.1. Droplet Morphology, Particle Size Determination and Size Distribution of Emulsions

**Figure 2:**
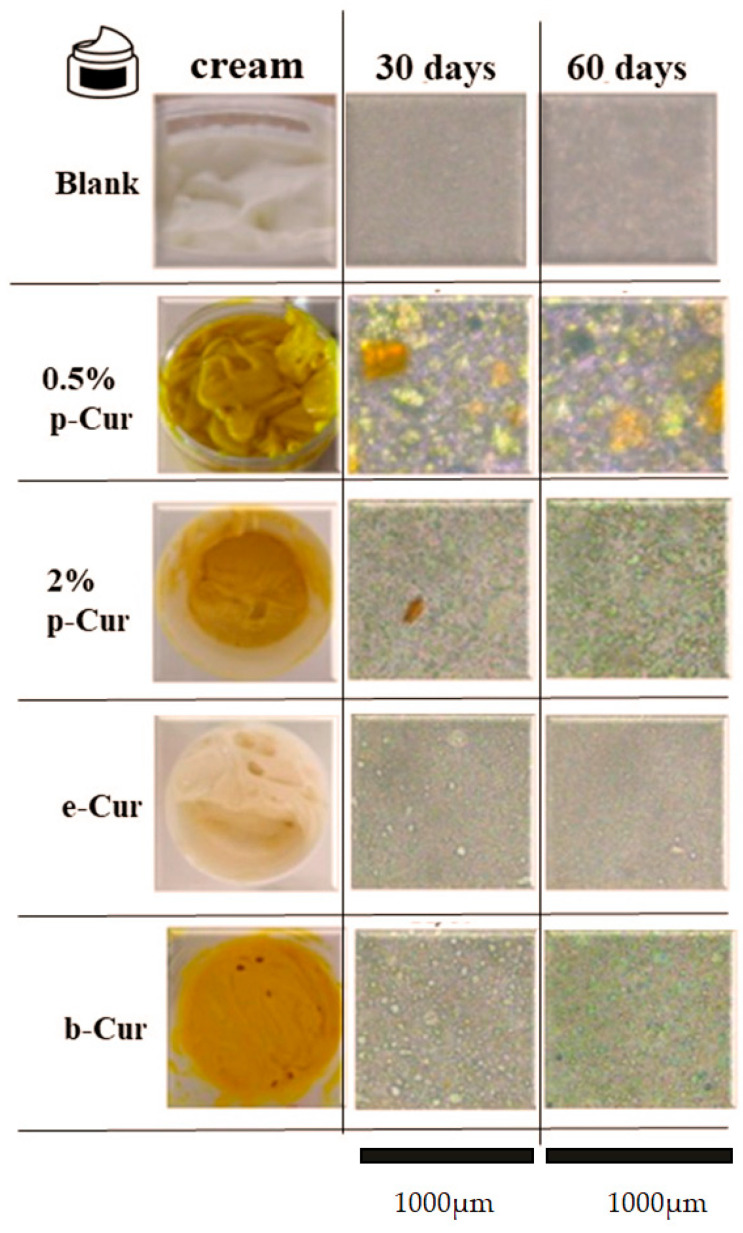
Optical microscope images of Blank, 0.5 and 2% *w*/*v* p-Cur, 1% *w*/*v* e-Cur and 1% *w*/*v* b-Cur emulsions, 1, 30 and 60 days after preparation and images of the prepared O/W emulsions using a ×20 lens.

Once an emulsion has been prepared, it is vital to remain stable and retain its initial properties throughout its storage time. Emulsions may breakdown due to various destabilization mechanisms including creaming, flocculation and coalescence [32]. The international literature proposes a variety of methods for studying the stability of emulsions [33,34,35,36,37]. Unfortunately, the instability affects their storage as they tend to separate and breakdown. In this context, macroscopic and microscopic tests to control the stability, viscosity and pH, as well as particle size distribution of the emulsions, are conducted. These include optical observation, pH and viscosity measurements, and estimation of the dispersed-phase droplet size density. In that sense, the particle size and the droplet stability of the prepared emulsions were investigated over a period of 60 days using an optical microscope. Table 2 presents the particle size and span for each emulsion, 30 and 60 days after preparation, respectively. Additionally, the images in Figure 2 demonstrate the optical analysis of each emulsion using a ×20 lens for the depiction of particle size of the internal phase. Specifically, blank emulsion presents a medium droplet size, stable until 60 days after fabrication, which was also confirmed by optical analysis. The obtained micrographs showed the formation of yellow solid agglomerates regarding the emulsions containing 0.5 and 2% p-Cur, with the ones at 0.5% emulsion being larger [38]. These results are attributed to the poor solubility and stability of p-Cur in aqueous media. However, the droplet size remains unaltered for up to 60 days after fabrication [37]. Results from microscope images of b-Cur emulsions exhibited smaller droplet size and high stability, since β-Cyclodextrin operates as a carrier of Cur, significantly enhancing its solubility and stability [15]. Moreover, the e-Cur emulsion contained droplets having smaller size but exhibiting higher stability than b-Cur emulsions, owing to the enhanced solubility of e-Cur [39]. Additionally, the span values of samples were calculated and studied considering that a monodisperse distribution has a span of 0 [40]. The results (Table 2) showed that the span of 0.5 % p-Cur emulsion was small (<2) during 30 and 60 days, presenting a narrow particle size distribution profile [41,42]. However, the addition of b-Cur, e-Cur and larger amount of p-Cur (2%) increased the span values, indicating a decrease in the homogeneity of emulsions.

In general, the emulsions with increased size of droplets in the internal phase are more unstable. This may be ascribed to the fact that droplets will grow in size, coalesce and form agglomerates, eventually leading in the destabilization of the emulsion. From the received results it is concluded that the emulsion containing e-Cur exhibits the highest stability.

### 3.2. Viscosity Stability

**Figure 3:**
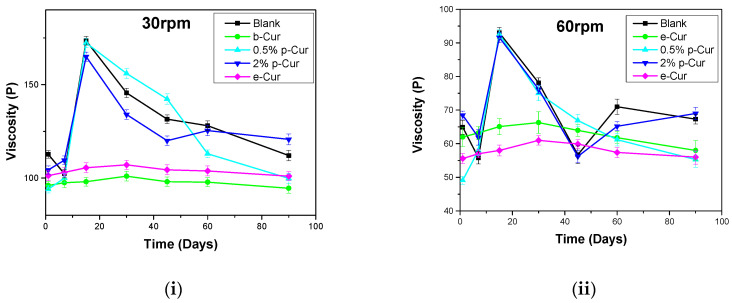

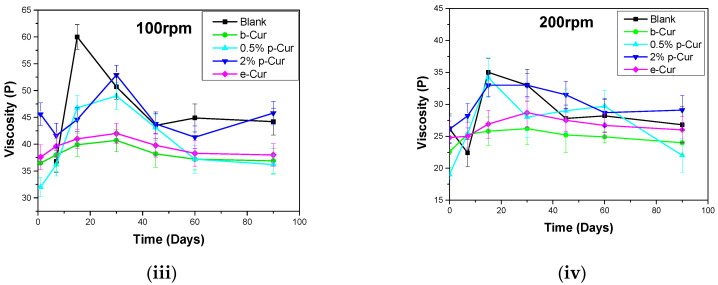
Viscosity profile for all emulsions at (**i**) 30 RPM (**ii**) 60 RPM (**iii**) 100 RPM and (**iv**) 200 RPM.

Another significant parameter concerning the emulsions’ stability is viscosity variation, since it is an indicator of their inner stability. Changes in viscosity during storage may result in several defects in the final formulations, such as phase separation and liquefaction, as well as alter their aesthetic appearance, which strongly affects the appeal of the end-product to the consumers [43]. Figure 3i–iv present the viscosity stability profiles of all prepared formulations at 30, 60, 100 and 200 RPM, respectively, during storage for up to 90 days. It is observed that the viscosity profile of all prepared emulsions decreased with increasing shear rate, presenting a distinct shear-thinning behavior as described by McClements et al. [44].

As RPM increases, droplet flocs or Cur agglomerates progressively deform and break down, thus decreasing flow resistance and viscosity. The produced emulsions exhibited different viscosity stability depending on Cur derivatives. In the case of p-Cur-based emulsions, the emulsions containing p-Cur either 0.5 or 2% wt showed the highest viscosity in 30, 60, 100 and 200 RPM. In particular, 0.5%and 2% p-Cur emulsions exhibited an increase higher than 10% in viscosity after 20 days and a decrease higher than 10% after 90 days of storage. This is a common behavior of emulsions containing agents in the form of powder, which leads in their flocculation. In the first stage, the agglomerates hinder the stirring, thus increasing the viscosity, while the following reduction in viscosity is attributed to the breakage of the agglomerates, producing more homogenous emulsions [45]. On the contrary, b-Cur and e-Cur emulsions showed an increase (less than 10%) in viscosity up to 30 days, and then a small decrease was observed up to 90 days (again less than 10% from the initial value), indicating that the prepared formulations show excellent performance in terms of stability. Similar results were observed in the case of 30, 60, 100 and 200 rpm [43]. In all these cases the particle size defines the viscosity stability of emulsions. The smaller the particle size, the more stable the viscosity profile. As the range of particle size becomes wider, numerous agglomerates are formed during the stirring and storage time, leading to viscosity instabilities.

### 3.3. pH Stability

The pH stability of the prepared emulsions is a crucial aspect, since changes in pH values indicate the occurrence of side chemical reactions. Given that the healthy human skin pH value varies from 4.5 to 6.0, cosmetic products intended for topical use should have a pH value that is within this range. Severe skin disorders may possibly occur from extreme pH values [4]. Therefore, in the current work, pH testing up to 90 days of storage was conducted for all fabricated emulsions. In all cases, the pH values were ranging within acceptable limits for human skin applications, i.e., from pH 5.2 to 5.9. However, it was found that the addition of p-Cur at 0.5 and 2% affect the pH stability of emulsions, presenting a bigger deviation from their initial pH value [17].

**Figure 4:**
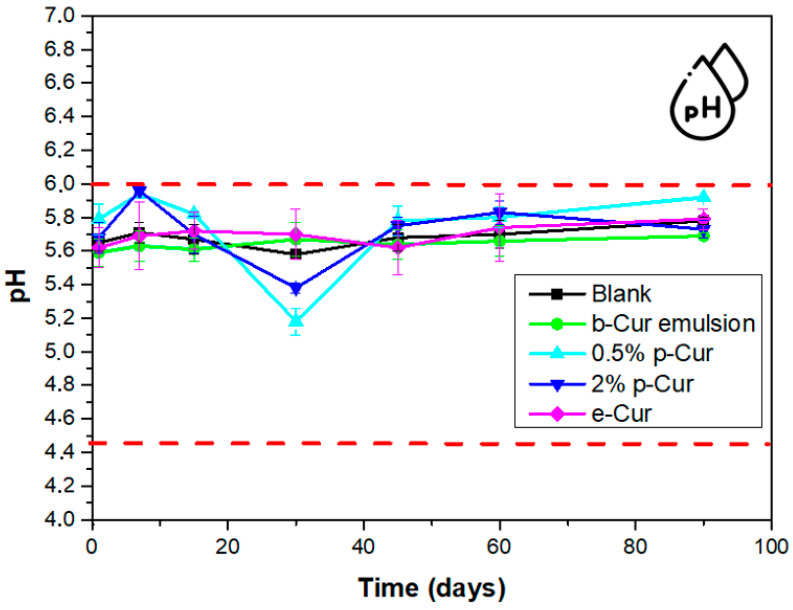
Change in pH values over time for all prepared O/W emulsions.

From Figure 4 it is observed that the emulsions with e-Cur and b-Cur do not affect the pH values over time. This was expected since the e-Cur and b-Cur emulsions presented small droplets which remain stable up to 90 days compared to emulsions with 0.5 and 2% *w*/*v* p-Cur which exhibited agglomerates and unstable morphology, also affecting the pH stability of emulsions. Therefore, from these measurements it can be concluded that e-Cur and b-Cur emulsions present steady pH values during storage, with no significant variations, making them suitable for cosmetic dermal applications.

### 3.4. Sunscreen Activity

**Figure 5:**
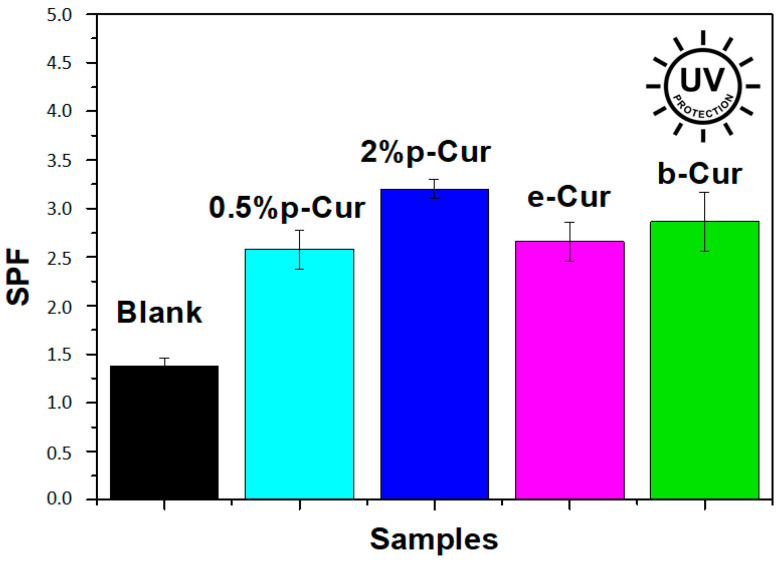
Sun protection factor (SPF) values of the prepared emulsions.

Exposure to UV light has a destructing effect on skin health, accelerating the process of photoaging, causing skin redness, premature aging, decreased skin elasticity and collagen synthesis. Consequently, sun protection constitutes a significant feature of cosmetic products. Curcumin is considered a great natural sunscreen agent, due to its active constituents, known as flavonoids. Several studies have shown that the flavonoids are quite beneficial for preventing UV-induced oxygen free radical generation and lipid peroxidation, i.e., events involved in pathological status, such as photoaging and skin cancer. The quantitative measurement of the effectiveness of a sunscreen is expressed by the sun protection factor (SPF). In order to be effective in preventing sunburn and other skin damage, a sunscreen product should have a wide range of absorbance between 290 nm to 400 nm [18]. Figure 5 presents the SPF values of the prepared emulsions compared to the blank. SPF values were calculated according to the Equation (1), and the absorption values were obtained in the range of 290–320 nm. All emulsions showed SPF factor values varying from 2.5 to 3.2 while the blank presents 1.3 SPF value, which is an indication that the addition of Cur and Cur derivatives improves SPF values. Furthermore, it was proved that by increasing the content of p-Cur, the SPF value increased (0.5% p-Cur and 2% p-Cur had 2.58 and 3.2 SPF values respectively). b-Cur emulsion presented a slightly higher SPF value than e-Cur emulsion while the 2% p-Cur emulsion presented the highest SPF value. According to FDA (U.S. Food and Drug Administration), any sunscreen agent utilized in sunscreen emulsions should display a minimum of SPF 2, and it is clear that all prepared emulsions meet this requirement [32].The obtained results demonstrated the ability of Cur, e-Cur and b-Cur to absorb UV radiation and hence proved their UV protection ability.

### 3.5. Antioxidant Properties

**Figure 6:**
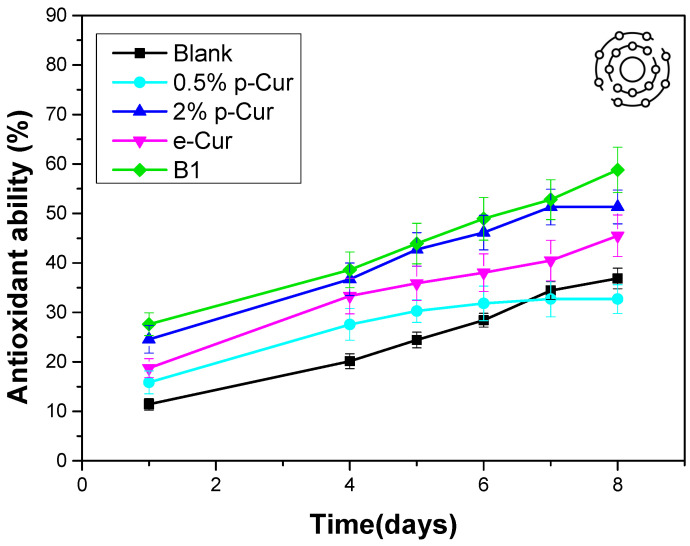
Κinetics of the antioxidant ability of emulsions during the first 8 days after preparation.

To counter the aging effects, the majority of consumers make use of skin products with antioxidant agents. Antioxidant agents are an important part of skin care formulations that prevent oxidative cell damage by acting as free radical scavengers. Free radicals (superoxide radicals [O_2_ radical dot−], hydroxyl radicals [OH radical dot] and singlet oxygen) are produced continuously in the human body by complex redox reactions and play a crucial role in the development of aging. Moreover, antioxidants are necessary ingredients in any emulsion to avoid the demulsification and quality deterioration occurred from lipid oxidation. Therefore, the addition of antioxidants to the emulsions and the study of their antioxidant ability is mandatory [4,33]. Curcumin has the capability of scavenging superoxide radicals, hydrogen peroxide and nitric oxide from activated macrophages. The major bioactive substances in Cur are polyphenols, including curcumin, which is well known, besides other polyphenols, for its excellent antioxidant activity [33]. During antioxidant studies, all samples presented enhanced antioxidant ability compared to the blank sample (Figure 6). This improved antioxidant activity of emulsions is attributed to the presence of the phenolic compounds due to the incorporation of p-Cur, e-Cur and b-Cur. There was a clear trend that the antioxidant capacity of the emulsions increased sharply with increasing content of p-Cur. When the p-Cur content was 2% *w*/*v*, the antioxidant capacity of the emulsion reached 51.3% while the antioxidant capacity of the 0.5% p-Cur emulsion was 32.7% [34]. This was expected as the phenolic compounds and curcumin content also increases (468 μg/mL at 2% p-Cur and 286 μg/mL at 0.5% p-Cur emulsion). However, although the b-Cur emulsion exhibits the highest antioxidant activity, the content of b-Cur antioxidant agent in the emulsion is 1% *w*/*v* is worth mentioning. This is explained by the fact that b-Cur presents the highest contain of phenolic compounds and curcumin (535 mg/mL) as it was proved by LC-HRMS Orbitrap Analysis (the results of method are presented in supplementary). This indicates that the b-Cur is more effective than both p-Cur and e-Cur (curcumin content in e-Cur was 321 μg/mL). These results are in total accordance with Murali MohanYallapu’s study of the in vitro antioxidant activity of b-Cur against HepG-2 cells that confirmed its enhanced antioxidant effect compared to neat e-Cur [15]. Furthermore, it is observed from Figure 6 that all emulsions with Cur compounds presented a progressive increase in the % antioxidant capacity up to 8 days after their preparation. This was also observed by Pilar Almajano’s working group that studied the synergistic effect of bovine serum albumin (BSA) on model oil-in-water emulsions containing a variety of green tea catechins. They found out a progressive increase in antioxidant activity for up 31 days of study [46]. This may be attributed to the synergistic effect taking place during storage between curcumin compounds and olive oil used in the oil phase of the prepared emulsions [47]. 

### 3.6. In Vitro Antimicrobial Studies

The assurance of antimicrobial quality in cosmetics is normally approached by the addition of preservatives that maintain their microbiological purity during manufacture, packing, storage, but especially during the entire period of usage [35].However, preservatives are considered as one of the main factors inducing allergies to users [36].To overcome these drawbacks, numerous approaches have been focused on the discovery of potent bioavailable and biocompatible ingredients with antimicrobial properties, such as polymer agents, chitosan [1]; herbal extracts, aloe vera [37] and calendula [48]; and essential oils, Lavandulla [49] and cinnamomum [50].

**Figure 7:**
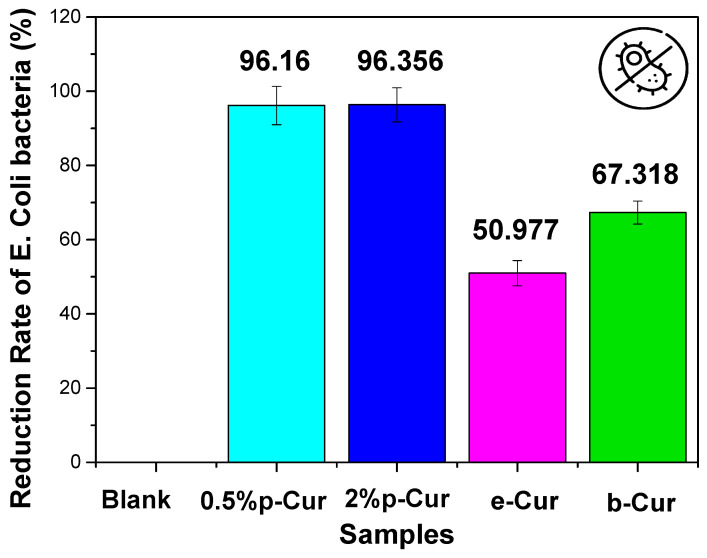
The reduction rate of investigated Cur emulsions against *E. coli* bacteria.

**Figure 8:**
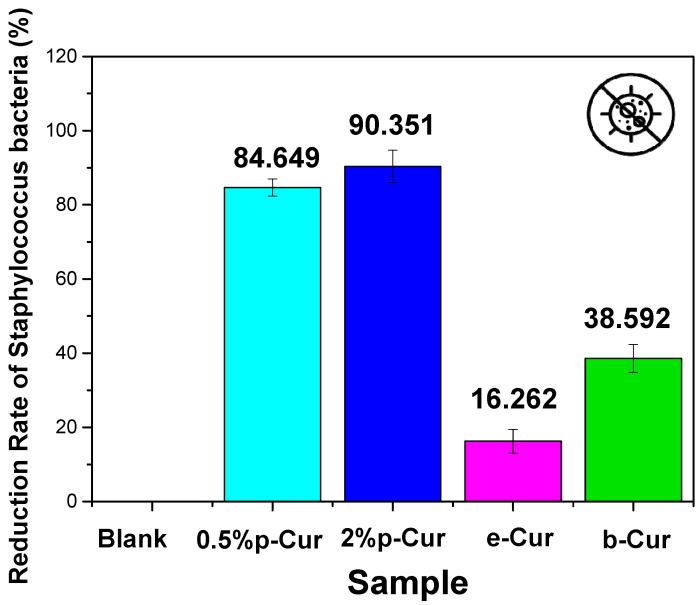
The reduction rate of investigated Cur emulsions against *Staphylococcus* bacteria.

Cur is amongst the herbs that exhibit both antibacterial and antioxidant properties. It is enriched with biological active compounds such as vitamin C, E, alkaloid, curcumins, turmerol and valeric acid, that have proven therapeutically antimicrobial effect [51]. Assorted studies have shown that Cur and its derivatives can be used for the treatment of various diseases since it exhibits antibacterial, anti-inflammatory, antiviral, antifungal, antiangiogenic, antioxidant and anticancer properties [52]. Numerous studies have reported that Cur extract successfully inhibited the growth of *Escherichia coli*, *Staphylococcus aureus* and *Candida albicans* [53]. Specifically, previous studies proved that the minimum inhibition concentration (MIC) of curcumin against *Staphylococcus aureus* and *E. coli* was 187.5 μg/mL and 163 µg/mL, respectively [54,55] Herein, Cur emulsions (Cur 0.5%, Cur 2%, e-Cur and b-Cur) were studied in terms of efficiency against two different bacteria, *E. coli* and *S. aureus* (Figure 7 and Figure 8). The two selected bacterial strains are the main constituents of wound infections due to their increased resistance to different classes of antibiotics [56]. The current results showed that the addition of Cur and its derivatives significantly decreased the bacterial count of two strains compared to the control group, in different concentrations. These results were expected since the curcumin concentrations (468 μg/mL at 2% p-Cur, 286 μg/mL at 0,5% p-Cur, 535 mg/mL at b-Cur, and 321 μg/mL e-Cur) of the prepared emulsions are above the MIC level, and, thus, it is expected to exhibit antimicrobial properties. Additionally, the results of the current study revealed that by increasing of p-Cur concentration, the antimicrobial property of the emulsions was enhanced. However, the reduction rate of bacteria did not decrease significantly against the two colonies; thus, the reduction rate of investigated p-Cur emulsions appears to reach a maximum value and remains almost steady.

On the contrary, the b-Cur complex, although containing the highest curcumin concentration, showed in both cases a lower percentage of anti-microbial activity against *E. coli* and *Staphylococcus* (67% and 38.5%, respectively) compared to the aforementioned 0.5% p-Cur and 2% p-Cur emulsions. This might be attributed to the development of resistance of staphylococcical strains to semisynthetic beta-lactamase-resistant compounds (i.e., oxacillin, methicillin and dicloxacillin) as has been previously reported by Vollono et al. [57].

Similar behavior was also observed in the e-Cur emulsion which showed the lowest antimicrobial capacity amongst the two colonies: 50.9% to *E. coli* and 16.2% to *Staphylococcus* bacteria. This is probably attributed to the processing techniques employed for the synthesis of e-Cur and b-Cur. According to Shirsath et al., the techniques that are used for the solvent extraction of natural products are usually associated with long extraction times and poor efficiency caused by used high temperatures [58]. As a result, a plethora of such products that are thermally unstable may be degraded during the procedure [14,59,60]. The results presented herein coincide with Chandarana’s study, claiming that Cur is active against *E. coli*, *B. subtilus* and *S.aureus* owing to its phenolic compounds, such as curcuminoids.

## 4. Conclusions

In the present study, O/W emulsions containing 0.5 and 2% *w*/*v* p-Cur ratios and 1% *w*/*v* e-Cur and b-Cur ratios were prepared and extensively investigated. The images obtained from the Optical Microscope, as well as the DLS measurements, showed that p-Cur emulsions contain agglomerates produced during the O/W emulsion preparation, due to the low solubility of p-Cur powder in the lipid phase. On the contrary, e-Cur and b-Cur emulsions presented smaller droplets size, due to the improved solubility and stability of e-Cur and b-Cur in the lipid phase. Stability tests indicated great viscosity and pH stability for both e-Cur and b-Cur up to 90 days of storage at room temperature. In contrast, p-Cur emulsions showed instability with large variations in terms of viscosity and pH values, due to the formed agglomerates. Regarding the sun-protective ability, obtained SPF values varied between 2.6 and 3.2, with the highest SPF value being obtained by the emulsion containing 2% *w*/*v* p-Cur. The antioxidant study of the prepared emulsions demonstrated that all prepared emulsions with Cur and its derivatives exhibited excellent antioxidant activity associated with the existence of bioactive polyphenols in the Cur structure. Despite the fact that e-Cur and b-Cur emulsions exhibited high viscosity and pH stability, as well as an improved antioxidant activity, they presented a low percentage of antimicrobial ability against two bacteria stains, *E. coli* and *Staphylococcus* bacteria. On the contrary, the 0.5 and 2% p-Cur emulsions presented a reduction rate over 90% against the same bacteria colonies. Antioxidant and antimicrobial properties results were also enhanced by LC-HRMS Orbitrap Analysis and the quantification of the phenolic compounds. In future research, it would be interesting to investigate the potential of the prepared emulsions as dermal Cur delivery systems for the treatment of skin cancer.

## Figures and Tables

**Table 1:** Ingredients used for the preparation of O/W emulsions.

Sample Name	Blank	p-Cur 0.5%	p-Cur 2%	b-Cur 1%	e-Cur 1%
Ingredients (%)	Water Phase (75%)				
Water	70	69.5	68	69	69
Glycerin	3.5	3.5	3.5	3.5	3.5
Citric acid	0.5	0.5	0.5	0.5	0.5
Xanthan gum	1	1	1	1	1
Cur		0.5	2		
β-cyclodextrin Cur				1	
Cur extract					1
	**Oil Phase (25%)**				
Olive oil	13	13	13	13	13
Cetyl alcohol	2	2	2	2	2
Cetearyl alcohol	2	2	2	2	2
Steatic acid	2	2	2	2	2
Shea butter	2	2	2	2	2
Beeswax	2	2	2	2	2
Polysorbate 60	2	2	2	2	2

**Table 2:** Particle size and distribution after 30(a) and 60 (b) days of fabrication for all emulsions.

Sample	^a^ D (v, 0.1) μm	^b^ D (v, 0.1) μm	^a^ D (v,0.5) μm	^b^ D (v, 0.5) μm	^a^ D (v, 0.9) μm	^b^ D (v, 0.9) μm S	^a^ Span	^b^ Span
Blank	10.77 ± 1.2	9.06 ± 1.3	53.07 ± 4.6	52.12 ± 5.1	203.80 ± 12.3	199.76 ± 8.2	3.63	3.65
0.5% p-Cur	20.04 ± 1.8	20.40 ± 2.3	82.18 ± 6.3	81.70 ± 5.7	168.58 ± 9.2	168.30 ± 7.1	1.80	1.81
2% p-Cur	14.36 ± 2.1	12.87 ± 1.5	51.85 ± 3.8	49.62 ± 4.9	158.30 ± 9.8	146.22 ± 6.8	2.77	2.68
b-Cur	1.32 ± 0.1	1.33 ± 0.13	5.29 ± 0.9	7.76 ± 1.5	29.77 ± 2.1	30.98 ± 2.9	5.37	3.82
e-Cur	1.46 ± 0.09	1.54 ± 0.11	3.58 ± 0.4	3.32 ± 0.96	15.30 ± 1.5	14.97 ± 1.2	3.86	4.04

D (v, 0.1), denotes the size of particle for which 10% of sample particles are smaller than this size. D (v, 0.5), denotes the size of particle for which 50% of sample particles are smaller than this size. D (v, 0.9), denotes the size of particle for which 90% of sample particles are smaller than this size. ^a^ D denotes the size of particle in 30 days. ^b^ D denotes the size of particle in 30 days. Span = (d(v, 0.9) − d(v, 0.1))/d(v, 0.5).

## Data Availability

Not applicable.
